# Successful Treatment of Basaloid Squamous Cell Carcinoma of the Nasal Cavity With Brain Invasion

**DOI:** 10.7759/cureus.35293

**Published:** 2023-02-22

**Authors:** Rieko Ii, Masahiro Nakayama, Shuho Tanaka, Hiroyoshi Akutsu, Keiji Tabuchi

**Affiliations:** 1 Department of Otolaryngology - Head and Neck Surgery, University of Tsukuba, Tsukuba, JPN; 2 Department of Neurosurgery, Dokkyo Medical University, Mibu, JPN

**Keywords:** 5 year survival, brain invasion, combined surgery, proton beam therapy, induction chemotherapy, basaloid squamous cell carcinoma

## Abstract

Basaloid squamous cell carcinoma (BSCC) is a rare and aggressive subtype of squamous cell carcinoma (SCC). To date, no consensus on the treatment of BSCC has been established yet, especially in cases of invasion of the skull base. In addition, long-term prognosis has not been reported in T4b cases. Herein, we report the case of a 36-year-old Japanese man with locally advanced nasal BSCC that directly invaded the skull base and the brain. The patient was then treated with induction chemotherapy (IC). Owing to his good response to IC, we planned and performed en bloc resection followed by adjuvant proton beam therapy (PBT). Follow-up examinations five years after treatment showed no evidence of recurrence. This is the first report of IC followed by radical surgery and adjuvant PBT in a patient with T4b. IC has the potential to play an important role in treatment strategies.

## Introduction

Basaloid squamous cell carcinoma (BSCC) is a rare variant of squamous cell carcinoma (SCC). In 1986, Wain et al. [1] first reported that the histopathological characteristics of BSCC are distinct and specific. Since the first report, only 65 cases of BSCC of the nasal cavity or paranasal cavity have been reported in the English literature identified through a PubMed search [2-9]. Various treatment modalities have been reported, including surgery supplemented with postoperative radiotherapy/adjuvant chemotherapy [2-9]. There is no established consensus on the treatment of BSCC because of the limited number of cases, especially in patients with T4b with an invasion of the skull base. In T4b cases of BSCC, only five cases have been reported, but reports of long-term prognosis or induction chemotherapy (IC) are missing [4,6-8]. Herein, we report the case of a 36-year-old man who was successfully treated with IC followed by radical surgery and adjuvant proton beam therapy (PBT) for locally invasive BSCC of the nasal cavity.

## Case presentation

**Figure 1:**
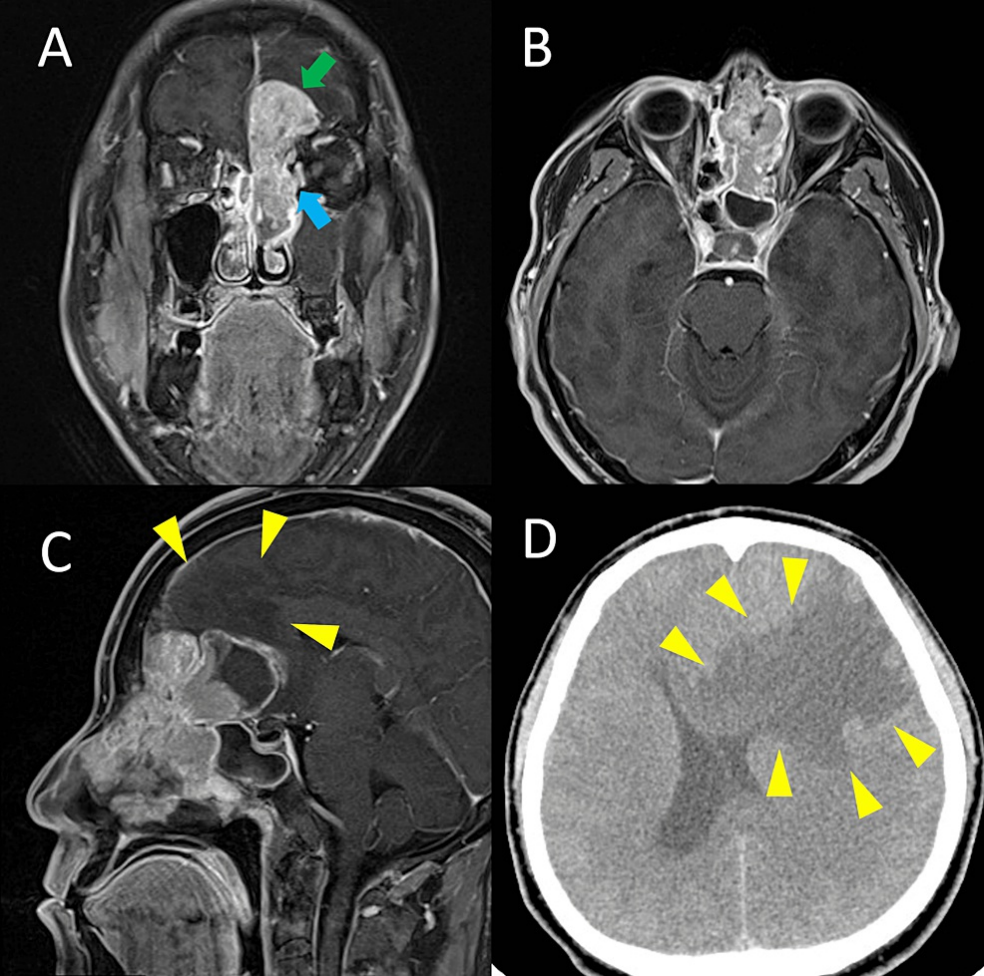
Imaging findings at initial examination and seven days after the initial examination Contrast-enhanced MRI shows the large tumor occupying the left nasal cavity with extension into the ethmoid sinus, frontal sinus, and sphenoid sinus [coronal(A), axial (B) and sagittal (C) plane]. The tumor extends to the frontal lobe (A: green arrow), exerting local pressure on the medial orbital wall (A: blue arrow) and infiltrating the anterior skull base. There is large perifocal edema in the frontal lobe (C: yellow arrowhead). (D) CT image seven days after the initial examination shows further progression of the brain edema (D: yellow arrowhead).

**Figure 2:**
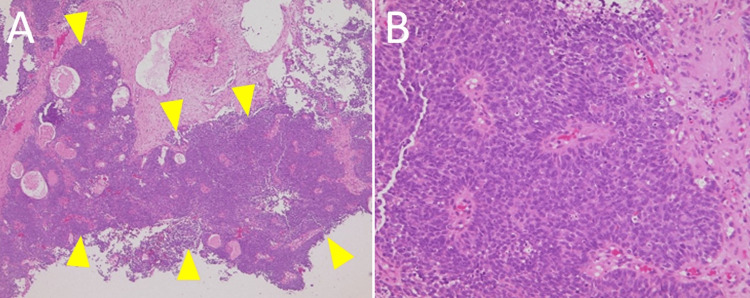
Histopathological findings (A) Hematoxylin and eosin (HE) staining (×40): The yellow arrowhead shows a malignant cell area. The tumor is composed of closely packed solid lobules of basaloid cells. There are also intercellular hyaline globules and necrotic foci are scattered. (B) HE staining (×200): The tumor has hyperchromatic nuclei with a high nuclear-to-cytoplasmic ratio. The peripheral palisading of the nuclei is seen. In some parts, it is a cord-like pattern.

A 36-year-old man presented to our hospital in 2017 with a two-month history of left nasal obstruction and headache. Upon clinical examination, the patient’s left nasal cavity was filled with a hemorrhagic tumor, and a biopsy was performed at the initial visit. Computed tomography (CT) and magnetic resonance imaging (MRI) revealed a large tumor extending into the left nasal cavity, ethmoid sinus, frontal sinus, and sphenoid sinus. The tumor extended into the frontal lobe, and there was severe edematous change. The tumor measured 57 mm × 32 mm × 55 mm (Figures 1A-1C). There was no clinical evidence of cervical or distant metastasis or neurological deficit. Seven days after the first visit, the patient was dazed. Although we initiated treatment for pain and progressive brain edema, his consciousness was rapidly impaired. The Glasgow Coma Scale score was E3V3M5 two weeks after the first visit. A CT scan showed further progression of brain edema (Figure 1D). Histopathological findings revealed high-grade carcinoma with clear cell, basaloid, and squamous features. The diagnosis was most consistent with BSCC (Figures 2A, 2B). The tumor was staged as T4bN0M0 stage IVB (Union for International Cancer Control [UICC] TNM system [8th edition]).

**Figure 3:**
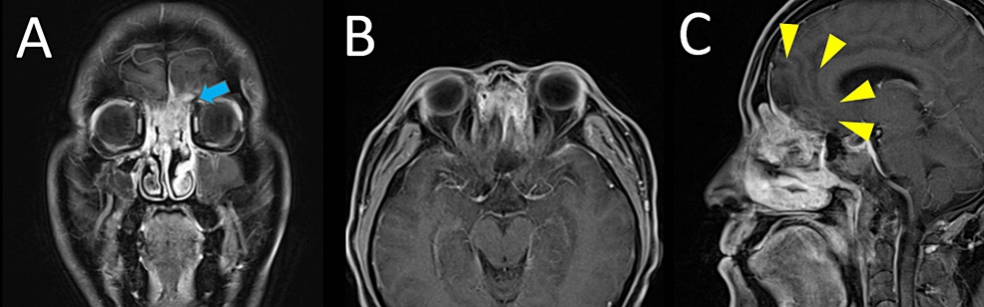
Contrast-enhanced MRI after three cycles of IC Coronal (A), axial (B), and sagittal (C) planes: the tumor decreased in size from 67 mm × 32 mm × 55 mm to 38 mm × 28 mm × 33 mm. The abnormal enhancing areas in the frontal lobe white matter also tended to disappear (C: yellow arrowhead). The tumor had extensive contact with the left frontal base (A: blue arrow).

This case was initially considered an unresectable tumor due to extensive invasion of the skull base and brain, with progressive brain edema. Although the patient had impaired consciousness, other physical examinations, including renal and liver functions, were normal. Therefore, the patient received IC with TPF (docetaxel, cisplatin, and 5-fluorouracil) eight days after the onset of consciousness disturbance. Owing to the low-performance status due to consciousness disturbance, a low-dose regimen of TPF (docetaxel at doses of 55 mg/m^2^; cisplatin at doses of 55 mg/m^2^/day on day 1; and 5-fluorouracil at doses of 550 mg/m^2^/day on days 1-5) was administered. The patient regained consciousness after the first IC cycle. After three cycles of TPF chemotherapy, his consciousness improved, and a partial tumor response was achieved (Figures 3A-3C). After IC, the tumor was removed with a combined endoscopic endonasal approach (EEA) and transcranial approach, as described in the surgical procedure section.

**Figure 4:**
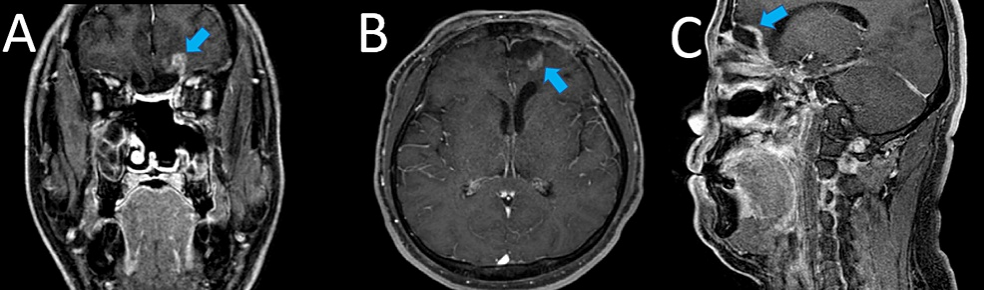
Contrast-enhanced MRI 13 months after the first treatment Coronal (A), axial (B), and sagittal (C) planes of MRI showed enhanced lesions in the left frontal lobe (blue arrow).

The patient was discharged without any complications two weeks after the surgery. The final histopathological examination revealed poorly differentiated BSCC with negative resection margins. Adjuvant proton beam therapy (PBT) was initiated 41 days postoperatively. The tumor bed region received a total radiation dose of 60 Gy (relative biological effectiveness) in 30 fractions. Thirteen months later, MRI showed an enhanced lesion in the left basal frontal lobe, which was suspected to be a recurrence or radiation necrosis (Figures 4A-4C). As the lesion grew after three months, it was removed via the transcranial approach. A histopathological examination revealed radiation necrosis. The patient has remained free from tumor recurrence for five years after PBT. He returned to work and is still working, as before.

Surgical procedure of combined EEA and transcranial approach

Combined surgery with EEA and a transcranial approach was performed by an interdisciplinary team of neurosurgeons and otolaryngologists at our hospital. Using EEA, we resected the total nasal bone, partial frontal bone, skull base bone, and medial orbital wall, as well as the tumor attachment in the sinonasal cavity. The resection margin was pathologically confirmed as negative. A neurosurgeon performed the transcranial approach using a bicoronal skin incision. The dural resection area was between the bilateral medial wall of the orbit on the coronal plane and between the anterior edge of the cribriform plate and the planum of the sphenoid bone in the sagittal plane. The tumor invading the frontal lobe, including the surrounding gliotic layer, was also resected via the transcranial approach. The resection margin was pathologically confirmed as negative. En-bloc resection was achieved, and the tumor was removed from the transcranial side. The skull base was reconstructed in a multi-layer fashion, with an in-lay sutured fascia lata on the frontal base dura reinforced with a pedicled pericranial flap fixed on the bone edge and was conducted from the transcranial side. Subsequently, a pedicled nasoseptal flap was placed on the pericranial flap on the EEA side.

## Discussion

BSCC of the tongue, hypopharynx, and larynx was first described by Wain et al. [1] in 1986. BSCC is a rare variant of SCC characterized by clinically aggressive behavior and distinct histological features [10]. BSCC occurs most frequently in the head and neck region, with a predilection for the larynx, hypopharynx, tonsil, and base of the tongue [2]. In contrast, BSCC of the sinonasal tract is uncommon, accounting for only 5% of all cases of head and neck BSCC [3].

The optimal management of sinonasal BSCC remains unclear. This usually requires multimodal treatment. The initial treatment generally involves surgical resection. Adjuvant treatment, consisting of radiotherapy with or without chemotherapy, has also been recommended [6]. Takamatsu et al. [5] reported that PBT concurrent with cisplatin may be an option for locally invasive BSCC. In other areas of the BSCC, Shibata et al. [11] reported a case of a patient with recurrent esophageal BSCC, who was successfully treated using systemic chemotherapy containing 5-fluorouracil and cisplatin. However, there is no established consensus on the treatment of BSCC, especially in patients with T4b with the invasion of the skull base.

In T4b cases of sinonasal BSCC, only five cases have been reported [4,6-8]. Only one case was treated with a combined approach of surgery followed by adjuvant chemoradiotherapy, but long-term results have not been reported [6]. However, the use of IC for sinonasal BSCC has not yet been reported. To date, there are no studies on IC for sinonasal BSCC and long-term survival with chemoradiotherapy. For the treatment of patients with T4b nasal and sinonasal malignancies, Okano et al. [12] reported that IC with docetaxel, cisplatin, and S-1 (TPS), followed by PBT concurrent with cisplatin, was well tolerated and could reduce complications, with promising antitumor activity. Licitra et al. [13] performed a retrospective analysis of 49 patients with previously untreated resectable sinonasal carcinoma, including stages I, II, III, and IV of the disease. PFL (cisplatin, fluorouracil, and leucovorin) was performed as IC, followed by surgery and postoperative radiotherapy. The three-year overall survival rate was 69%. IC may be effective for sinonasal cancers.

Our case was initially considered an unresectable tumor due to invasion of the skull base and brain with progressive brain edema associated with a disturbance of consciousness. Considering the efficacy of IC in patients with sinonasal T4b [12,13], we performed IC with TPF according to the standard regimen of IC for head and neck carcinoma. IC as the initial treatment resulted in a good response for the tumor and improved the level of consciousness. As the long-term outcome of chemoradiotherapy is unknown for T4b cases of BSCC, and the tumor became resectable after IC, we performed en-bloc resection with pathologically free margins and skull base reconstruction. This is the first report of IC followed by radical surgery and adjuvant PBT in a patient with T4b. As the disturbance of consciousness was due to the growth of the tumor itself, IC played an important role in our treatment strategy. Chow et al. [14] reported on the quality of life (QOL) of sinonasal tumors. QOL after endoscopic surgery for sinonasal and anterior skull base tumors seems to improve within several months of surgery in both the benign and malignant tumor groups. The patient was discharged from the hospital with no problems and has survived for five years and is working as before.

The margin was pathologically negative; however, postoperative PBT was performed owing to its proximity. Our institute is the first to initiate PBT in Japan. The benefits of PBT include reduced toxicity through normal tissue sparing or improved disease control through treatment intensification [15]. In a meta-analysis of PBT for nasal cavity and paranasal sinus malignancies, Patel et al. [16] reported that PBT improved disease-free survival (relative risk [RR] 1.44, p = 0.045) and locoregional control (RR 1.26, p = 0.011) compared to intensity-modulated radiation therapy (IMRT). Dagan et al. [15] retrospectively reviewed 84 patients treated with primary (13%) or adjuvant (87%) PBT for sinonasal cancers and reported that the three-year local control and overall survival rates were 83% and 68%, respectively. They also reported a high local control rate of 90% after gross total resection and adjuvant PBT, which far exceeded the previously reported local control rate with conventional radiation therapy and IMRT. There has only been one report of sinonasal BSCC treated with PBT. Takamatsu et al. [5] reported good results with chemotherapy and PBT in patients with T3. Our case is the second case of BSCC treated with PBT. Regarding toxicity, the incidence of grade 1-2 brain necrosis, according to the Common Terminology Criteria for Adverse Events version 5.0, was reported to be 7% in patients undergoing PBT for nasal cavities and paranasal sinus malignancies [17]. The patient survived for five years without recurrence after treatment with PBT, although partial radiation necrosis was observed.

For advanced BSCC with brain invasion, as in our case, IC with TPF may be selected as the initial treatment, and if a favorable tumor response is achieved, en bloc resection followed by adjuvant PBT may be planned. Combined surgical techniques, surgical oncologic principles, and a team-based approach with neurosurgeons, radiation oncologists, pathologists, and medical oncologists are needed to successfully treat locally invasive sinonasal cancers, such as BSCC.

## Conclusions

We report a case of locally invasive sinonasal BSCC treated with multimodal therapy, including IC, radical surgery, and PBT. Among these, IC played an important role in the treatment strategy and resulted in the expansion of treatment modalities. Although a standard treatment for locally invasive sinonasal BSCC has not yet been established, further studies are needed to establish a treatment for BSCC. This treatment strategy may be an option for locally invasive BSCC.
